# Spinal cannabinoid receptor 2 activation alleviates neuropathic pain by regulating microglia and suppressing P2X7 receptor

**DOI:** 10.3389/fnmol.2023.1061220

**Published:** 2023-03-08

**Authors:** Yifan Zhou, Yaowei Xu, Jingjie Yang, Zhixiang Yu, Wenting Wang, Meng Yuan, Yiming Wang, Qian Bai, Zhisong Li

**Affiliations:** ^1^Department of Anesthesiology and Perioperative Medicine, Second Affiliated Hospital of Zhengzhou University, Zhengzhou, China; ^2^Institute of Neuroscience, Academy of Medical Sciences, Zhengzhou University, Zhengzhou, China

**Keywords:** neuropathic pain, cannabinoid receptor 2, microglia, P2X7 receptor, neuroinflammation

## Abstract

Neuropathic pain (NP) is the chronic pain in patients resulting from injuries or diseases in the somatosensory nervous system. However, effective treatment remains limited to opioids. Currently, there is an urgent need to develop new specific pharmaceuticals with low abuse potentiality. Cannabinoid receptor 2 (CB2R) is one of the significant receptors in the endocannabinoid system. It is widely expressed in the central nervous system, especially enriched in glial cells, and plays an important role in the occurrence and development of inflammation in the nervous system. CB2R activation has a neuroprotective effect on nerve injury. In this study, we report increased and more reactive microglia (with larger cell body, shorter processes, and fewer endpoints) observed in the spinal dorsal horn of spared nerve injury (SNI) rats. Continuous intrathecal administration of CB2R agonist PM226 attenuated mechanical and cold hyperalgesia in rats and prevented the transition of microglia to the proinflammatory stage. Thus, microglia transitioned into the neuroprotective stage. Meanwhile, the proinflammatory factors TNF-α and iNOS decreased, and the levels of anti-inflammatory factors Arg-1 and IL-10 increased. The content of P2X7 receptors in the spinal dorsal horn of rats increases with time after SNI. After continuous intrathecal administration of PM226, the content of P2X7 protein decreases significantly. The administration of P2X7 inhibitor A-438079 alleviated the mechanical hyperalgesia of rats, reduced the number of microglia, and decreased the content of P2X7. These results indicate that P2X7 is involved in the neuroprotective effect caused by CB2R activation. In conclusion, this study provides new insights into the neuroprotective mechanism of CB2R activation.

## Introduction

Neuropathic pain (NP) is pain induced by somatosensory nerve system injury or illness, characterized by hyperalgesia, allodynia, and spontaneous pain (Wen et al., 2022). According to an epidemiological study, the incidence of NP in normal communities is 6.9%–10%, accounting for 20–25% of chronic pain (van Hecke et al., 2014; Bouhassira, 2019). Given the significant impact, NP poses on the physical and psychological health of patients, it is seen as a heavy burden on society. The search for medications against this disease has never stopped. Between year 2000 and 2020, more attention was put on finding pain medications with low potential for abuse. Even so, the success rate of development programs for compounds with limited abuse potential was only 4.7%. In contrast, the development programs for compounds with significant misuse potential had a higher success rate of 27.8%. Furthermore, the success rate for treating NP was just 7.1% (Maher et al., 2022). Consequently, it is urgent to find an effective method to solve this clinical problem.

The endocannabinoid system consists of two types of cannabinoid receptors, CB1 receptor (CB1R) and CB2 receptor (CB2R) (including TRPV1, GPR151 and PPARs in a broad sense), endogenous ligands, mainly 2-arachidonoylglycerol (2-AG) and N-arachidonoylethanolamine (anandamide), as well as metabolism-related hydrolases monoacylglycerol lipase (MAGL) and fatty acid amide hydrolase (FAAH) (Maldonado et al., 2016; Zubrzycki et al., 2019). Neuropathic pain involves neuronal pathways and microglia, which can interact with neurons to regulate pain transmission under pathophysiological conditions. Independent studies have illustrated that both CB1R/CB2R agonists have the effect of alleviating NP (Maldonado et al., 2016; Shiue et al., 2017; Banister et al., 2019). Considering that CB1R is mainly expressed in the central nervous system, a previous study found that CB1R agonists can produce neuropsychiatric symptoms, which limits their wide application (Cristino et al., 2020). CB2R, which widely exists in the immune system and can regulate the activity of microglia. In the peripheral nerve transection model, a CB2R agonist, JWH015, can reverse the increase in CR3/CD11b and the decrease in ED2/CD163 in the spinal cord of rats, which can be offset by CB2R antagonists instead of CB1R antagonists (Romero-Sandoval et al., 2008). It was also observed that, sciatic nerve injury can cause hyperalgesia in the ipsilateral and contralateral paws in CB2R knock-out mice, and hyperalgesia and microglia activation were attenuated in mice overexpressing CB2R (Racz et al., 2008). However, it remains unknown whether and how CB2R agonists affect microglia expression in NP.

In NP models, microglia contribute to the initiation of mechanical allodynia or thermal hyperalgesia, increasing the shift from acute to chronic pain. Previous studies have shown that in the injured CNS, microglia can be polarized into two phenotypes: detrimental M1-like and neuroprotective M2-like, also known as classic type and alternative type. This simple dualism is now regarded as an outdated nomenclature, since microglia are not immutable and will present different functions depending on the context (Paolicelli et al., 2022). However, some microglia-associated molecules affect certain functional states, cell surfaces, and secreted molecules, such as CD86, TNF-α, and iNOS, which are associated with degeneration, whereas others, such as CD163, Arg-1, and IL-10, are associated with homeostasis maintenance (Kroner et al., 2014; Lan et al., 2017; Yong, 2022). The further release of proinflammatory factors aggravates the pathological process, and the therapeutic effect can be achieved by reversing this process (Zhang et al., 2020; Wang et al., 2021; Jiang et al., 2022). Activation of CB2R can reduce the release of proinflammatory factors (Wu et al., 2019); however, whether CB2R agonists can reduce neuropathic pain by influencing microglial alterations is a question worth exploring.

P2X7 receptor, a member of the purinergic receptor P2X family, is mainly expressed on microglia in the central nervous system. Moreover, several studies have revealed its vital role in NP treatment (Chen et al., 2017; Gui et al., 2020; Hu et al., 2020). When neuroinflammation occurs, the number of P2X7 will be enhanced. The inhibition of P2X7 can attenuate hyperalgesia and reduce astrocyte and microglia activation in the L5 dorsal horn (Hu et al., 2020). Furthermore, P2X7 produces analgesic effects by participating in the regulation of microglia.

In this study, continuous intrathecal administration of PM226 (a novel, highly selective CB2R agonist) (Luongo et al., 2010) was employed. Effects of P2X7 receptor modification in the spinal dorsal horn of spared nerve injury surgery (SNI) rats on microglia polarization were described. The results can provide new molecular insights into the therapeutic effects of CB2R activation.

## Materials and methods

### Animals

Male Sprague Dawley rats (200–250 g, 6–8 weeks old) were acquired from Sbeff Bioscience Co., Ltd., Beijing, China. The rats were housed in separate cages in a controlled condition (a 12-h dark/light cycle, a room temperature of 22–25°C, and a humidity level of 50–60%, and drinking water and food were freely available *ad libitum*) for 7 days prior to the test. All procedures were certified by the Ethics Committee of the 2^nd^ Affiliated Hospital of Zhengzhou University (No.2022055) and followed the National Institutes of Health’s Guide for the Care and Use of Laboratory Animals. All attempts had been made to decrease the number of animals used and minimize their suffering.

### Spared nerve injury surgery

We used SNI model as previously described (Decosterd and Woolf, 2000) to induce neuropathic pain. The rats were anesthetized with 2.5% isoflurane, followed by skin preparation and sterilization. An incision was made in the left hind limb of the rat, and the femoris biceps muscle was passively separated to make the sciatic nerve, and its three branches visible. The peroneal and tibial nerves were ligatured with 5–0 silk. The two nerves mentioned above were cut off at the distal end of ligation, and the 2–4 mm nerve stump was removed, whereas the sural nerve stayed untouched. As for the sham group, the nerves were exposed but not ligated nor truncated.

### Behavioral testing

Before each behavioral test, all the animals adapted to the test environment 3 days in advance, half an hour every day. All behavioral tests were completed by practitioners with no knowledge of the grouping and treatment of the rats.

#### Mechanical allodynia assessment

We used the mechanical withdrawal threshold (MWT) to measure the mechanical allodynia of rats. Before starting, all animals were placed on an elevated wire mesh floor separated by plastic cells and adapted for 30 min. The hind paw was activated by a series of von Frey filaments with a bending force of 1.4, 2, 4, 6, 8, 10, 15, and 26 g (Aesthesio, United States) successively, and each filament was applied vertically to the lateral skin of the hind paw for 5 s. Recordings were made when the hind paw retracted significantly or lifted off the floor. We employed the up-down approach (Chaplan et al., 1994) to calculate the MWT.

#### Cold allodynia assessment

The acetone test (Yoon et al., 1994) was performed to assess the behavioral reactions of animals to a cold stimulus. All rats are separated in individual cells on an elevated wire mesh floor. Acetone (100 μL) was lightly sprayed onto the lateral plantar surface by means of a syringe connected with blunt pipette tips, and the reaction was observed within 10 s after stimulation. Responses were divided into 4 rating levels: 0, no response or moving around; 1, brisk withdrawal or the paw flicking; 2, repeated paw flicking; 3, repetitive flicking and licking of the hind paw (Leite Ferreira et al., 2022). Acetone was applied three times for each paw with an interval of 5 min each time. Finally, the average value of three repetitions was taken as the acetone score.

### Intrathecal catheterization

The intrathecal (i.t.) catheterization was performed 5 days prior to the SNI surgery as previously described (Størkson et al., 1996). Under 2.5% isoflurane anesthesia, the rats were shaved and prepared for the operation. Then, about a 2 cm incision was made in the lumbar region’s midline. The PE10 polyethylene catheter (outer diameter 0.61 mm, inner diameter 0.28 mm) was implanted into the subarachnoid space after the intravertebral space between L5 and L6 had been exposed. The other end of the catheter was fastened to the head end for subsequent administration. The position of the catheter was confirmed by an i.t. injection of 2% lidocaine. Animals exhibiting any motor dysfunction were not used in the experiments.

### Drug administration

The CB2R agonist PM226 (GC50387, GLPBIO, United States) was dissolved in normal saline. Firstly, the following concentration gradients are set to confirm the optimal dose and interval of intrathecal injection: 0.2, 2, and 20 mM, according to the preceding studies (Gomez-Canas et al., 2016; Morales et al., 2016; Navarro et al., 2016). On the 3rd post-SNI operation day, rats in the SNI + PM226 groups received a single intrathecal injection of 15 μL of different concentrations of drugs. At the same time, the SNI + Vehicle group received only 15 μL vehicle. The next experiment was performed to investigate how continuous intrathecal administration of PM226 has an influence on NP. From 2 h before the surgery, the SNI+ PM226 group received 15 μL 2 mM PM226 every day.

The P2X7 receptor antagonist A-438079 (HY-15488, MedChemExpress, United States) was dissolved in normal saline. According to the previous study (Hu et al., 2020), for the SNI + A-438079 group, intrathecal infusions of 100 pmol of A-438079 dissolved in 10 μL saline were carried out on day 3 after the SNI surgery. The SNI + Vehicle group received saline infusions of the same volume.

### Western blot

The L4–L6 spinal dorsal horn were gathered onto ice immediately after the rats were sacrificed under pentobarbital sodium (i.p., 40 mg/kg) anesthesia. After adding cold RIPA lysis buffer (#CW2333, CWBIO, China) with 1% 100 mM phenylmethylsulphonyl fluoride (PMSF) (P0100, Solarbio, China), the sample was homogenized at 4°C. The samples were centrifuged in a low-temperature centrifuge at 4°C for 15 min at 3,000 r/min, and then the supernatants were obtained. The BCA Protein Assay Kit (PC0010, Solarbo, China) was used to determine the concentration of protein. The samples were prepared to the same concentration and then heated to 99°C for 5 min. Similar quantities of proteins (20 μg) were segregated on 10% SDS-polyacrylamide gels and transported electrophoretically to PVDF membranes. After being blocked by 3% skim milk powder for an hour, membranes were treated with the primer antibody at 4°C overnight. The membranes were then treated with HRP-conjugated secondary antibodies for 2 h at ambient temperature. The bands were imaged by a ChemiDocTM machine (Bio-Rad, United States) using enhanced chemiluminescence (BL520A, Biosharp, China). Integrated optical density was quantified by Image J. The antibodies used above are listed in Table 1.

**Table 1:** The antibodies for western blot.

Antibody	Source	Catalog number	Dilution
CB2	Thermo Fisher Scientific, United States	PA1-746A	1:500
Iba1	ABclonal, Wuhan, China	A19776	1:1,000
TNF-α	ABclonal, Wuhan, China	A11534	1:2,000
CD86	Bioss, Beijing, China	bs-1035R	1:1,000
CD163	Bioss, Beijing, China	bs-2527R	1:1,000
Arg-1	Proteintech, Wuhan, China	16001-1-AP	1:5,000
P2X7	Affinity Biosciences, United States	AF4626	1:1,000
β-Tubulin	Servicebio, Wuhan, China	GB11017	1:5,000
GAPDH	Proteintech, Wuhan, China	60004-1-Ig	1:5,000
Goat Anti-Rabbit IgG	Proteintech, Wuhan, China	SA00001-2	1:5,000
Goat Anti-Mouse IgG	Proteintech, Wuhan, China	SA00001-1	1:5,000

### Immunofluorescence

Rats under deep anesthesia were perfused with 0.1 M phosphate buffer (PBS) and 4% paraformaldehyde (PFA) afterwards *via* the ascending aorta. The L4-6 spinal cord was collected and postfixed in the same fixative overnight at 4°C then replaced with 20, 30% sucrose in 0.1 M PBS, respectively, overnight. In a cryostat, transverse free-floating spinal cord pieces (25 μm) were sliced (Leica, CM1950). The sections were treated for 5 min with antigen retrieval solution (C1035, Solarbio, China), then blocked for 1 h at room temperature with 5% albumin bovine serum in 0.3% Triton X-100, followed by overnight at 4°C with the primary antibody. Afterward, the slices were treated for 2 h at room temperature with the matching secondary antibody. For double immunofluorescence staining, spinal segments were incubated with a combination of two primary antibodies (from two different species) and then processed with a mixture of two second antibodies. The staining of the slices was imaged by ECLIPSE Si (Nikon, Japan). The detailed information on the antibodies used above is listed in Table 2.

**Table 2:** The antibodies for immunofluorescence.

Antibody	Source	Catalog number	Dilution
CB2	Thermo Fisher Scientific, United States	PA1-746A	1:100
Iba1(Rabbit)	ABclonal, Wuhan, China	A19776	1:200
Iba1(Mouse)	Abcam, United Kingdom	Ab283319	1:2,000
CD86	Bioss, Beijing, China	bs-1035R	1:100
CD163	Bioss, Beijing, China	bs-2527R	1:100
P2X7	Santa Cruz, Dallas, TX, USA	sc-514,962	1:100
Goat anti-mouse Cy3	Jackson ImmunoResearch, United States	115-165-003	1:200
Goat anti-rabbit FITC	Jackson ImmunoResearch, United States	115-095-003	1:200

### Morphological examination of microglia

Iba1-immunostained L4-L6 spinal cord sections (40 μm, 3 sections from each rat) at 60× magnification were used to perform the morphometric analysis of microglia. The z-stacks with 1 μm interval between images of the sections were acquired by means of laser scanning confocal microscopy (A1 MP+, Nikon). As described previously (Young and Morrison, 2018), the images were set up to 8-bit and converted to grayscale to best visualize all microglia processes. After erasing all overlapping and incomplete cells, the solidity analysis (cell body area vs. processes area) was performed on the remaining cells using Image J (Roblain et al., 2021). To collect skeleton pictures for a quantitative study of average endpoints and process length, the AnalyzeSkeleton (2D/3D) plugin was utilized (Ma et al., 2020). For statistics, the averages value of the three sections were taken from each rat.

### Quantitative real-time PCR

In brief, as directed by the manufacturers, total RNAs from the L4-6 spinal dorsal horn were isolated using the TRIzol Reagent (DP419, TIANGEN Biochemical Technology, Beijing, China). cDNA was acquired by means of PrimeScipt RT Master Mix (RR047A, TaKaRa, Japan) at 37°C for 15 min and 85°C for 5 s. 20 μL of qPCR reactions were prepared using Premix Ex TaqII (RR420A, TaKaRa, Japan), then run on an Applied Biosystems StepOnePlus Real-Time PCR System (Foster City, CA, United States). The mRNA expression was measured by means of the 2-ΔΔCT, and standardized to a single control group. The primer sequences (designed by Sangon Biotech, Shanghai, China) used in the experiment are presented in Table 3.

**Table 3:** The primers for qRT-PCR.

Gene	Forward	Reverse
TNF-α	CTCCCAGAAAAGCAAGCAAC	CGAGCAGGAATGAGAAGAGG
CD86	AATCCTTTTCTCGGTGTTGG	CTCGGGCTTATGTTTTGAGC
iNOS	CCTGGTGCAAGGGATCTTGG	GAGGGCTTGCCTGAGTGAGC
Arg-1	TATCGGAGCGCCTTTCTCTA	ACAGACCGTGGGTTCTTCAC
CD163	TGGGCAAGAACAGAATGGTT	CCTGAGTGACAGCAGAGACG
IL-10	TAAGGGTTACTTGGGTTGC	TATCCAGAGGGTCTTCAGC
GAPDH	GCATCTTCTTGTGCAGTGCC	GATGGTGATGGGTTTCCCGT

### Statistical analysis

All data are presented as mean ± standard deviation (SD) and processed by GraphPad Prism 8 (Graph Pad Software, San Diego, CA, United States). ANOVA with two-way repeated measurements, followed by Bonferroni’s *post hoc* test, was employed in the analysis of the behavioral tests. Two-tailed t-test or one-way ANOVA processed by Bonferroni’s *post hoc* test was selected to analyze the data of WB, qRT-PCR and immunofluorescence. *p* < 0.05 was deemed statistically significant for all analyses.

## Results

### SNI changed the number and morphology of microglia in the spinal dorsal horn of rats

**Figure 1:**
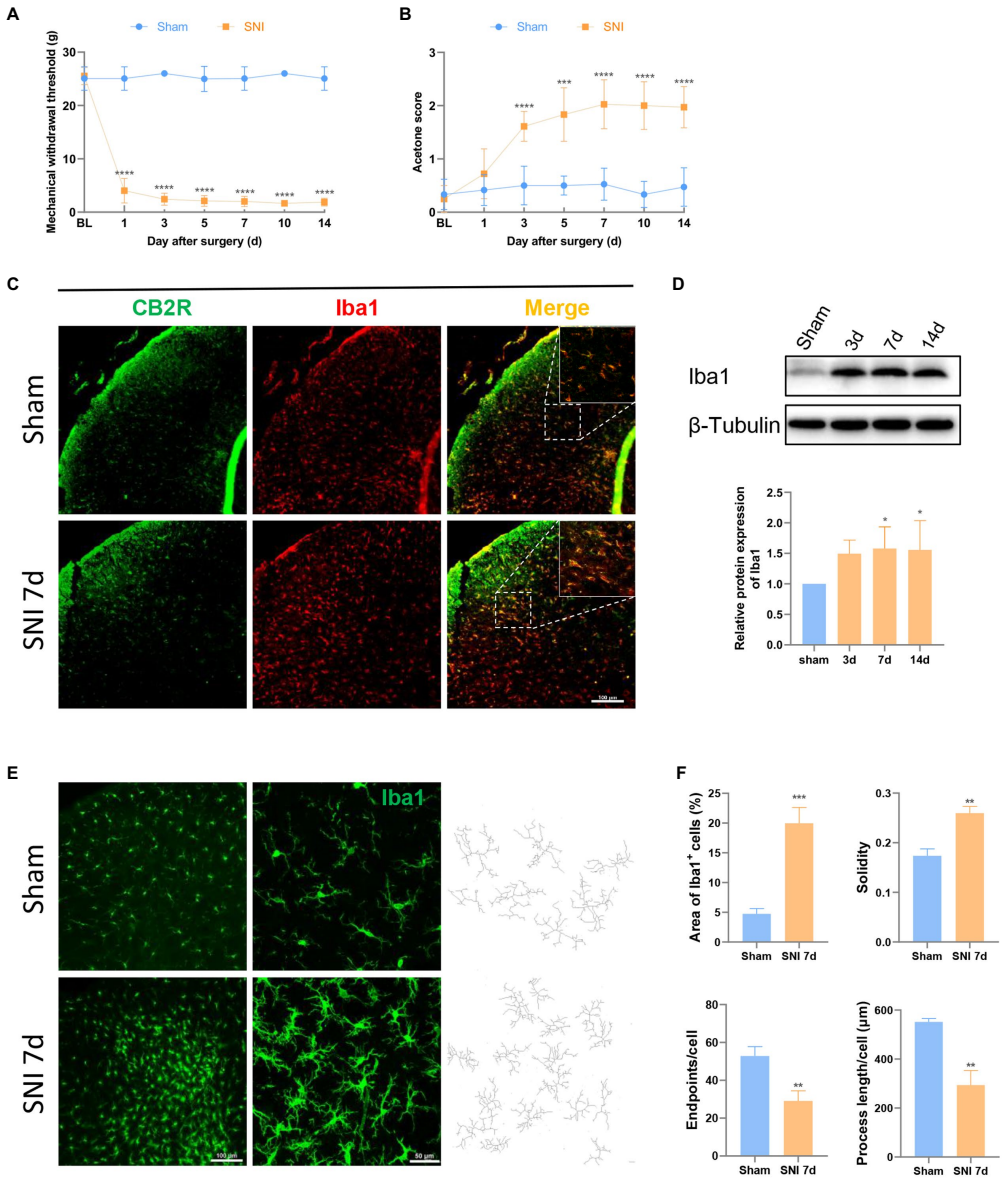
SNI induced hyperalgesia changed the expression and morphology of microglia in the spinal dorsal horn. **(A,B)** SNI significantly reduced the MWT **(A)** and improved the acetone score **(B)** in the ipsilateral paws. (****p < 0.0001 compared with the sham group, *n* = 12 in each group). **(C)** Double immunofluorescence of CB2R and Iba1 in the spinal cord of sham and SNI-7d rats. The fluorescence images in the boxes were magnified to better display the double staining. Scale bar, 100 μm. **(D)** The expression of Iba1 increased on days 3, 7, and 14 after SNI. (*p < 0.05 compared with the sham group, *n* = 6 in each group). **(E)** microglia micrographs from the ipsilateral spinal dorsal horn sham or SNI-7d rats. The images with higher magnification were skeletonized and expressed as black-and-white images (right side) for measuring the three parameters. Scale bars, 100 μm for lower magnification and 50 μm for higher magnification. **(F)** Quantification of the area of Iba1+ cells and morphology of microglia. Solidity, the area of cell body / the area of processes for each cell. Results for each group were obtained from three rats and three micrographs from each sample. The average values of each sample were taken for statistics. (**p < 0.01, ***p < 0.001 compared with the sham group).

Compared with the rats in the sham group, MWT decreased, and the acetone score increased after the SNI operation, which was statistically significant from day 3 and then stabilized posteriorly (Figures 1A,B). Similar phenomena were also observed on the ipsilateral and contralateral sides of SNI rats (Supplementary Figure 1A), indicating the success of SNI modeling. Furthermore, SNI surgery did not affect the pain sensitivity of the contralateral hindpaws, which showed no significant difference in MWT and acetone scores between the sham and SNI groups (Supplementary Figure 1B).

CB2R is a 7-transmembrane domain receptor coupled to inhibitory G proteins ubiquitously present in immune cells. Microglia could be observed in the spinal dorsal horn in the sham group and the SNI 7d group. Double immunofluorescence staining revealed CB2R exists in most microglia (Figure 1C), which was consistent with that was observed in other experiments (Racz et al., 2008; Wang et al., 2020). This is the structural basis for CB2R regulation of microglia.

After peripheral nerve injury, microglia respond immediately, showing increased numbers and morphological changes, and participate in the process of NP through their motility, release of soluble factors, and phagocytic ability, which is consistent with the observed results. On days 3, 7, and 14 after SNI, the protein content of the microglial marker Iba1 protein increased in the spinal dorsal horn of rats (Figure 1D). On days 7 and 14, the difference was obvious. This phenomenon was more intuitively reflected in immunofluorescence (Figure 1E). Under homeostatic conditions, microglia show long branches and small cell bodies. In the state of injury, inflammation, or other cases, the cell body increases, and the branches become shorter and fewer, which is called the “activated state” (Cao et al., 2021). Semi-automatic quantitative morphometric measurements of microglia revealed that the processes became shorter and branchpoints decreased 7 days after SNI compared to the sham group (Figures 1E,F).

### Microglia trended to proinflammation stage after SNI

**Figure 2:**
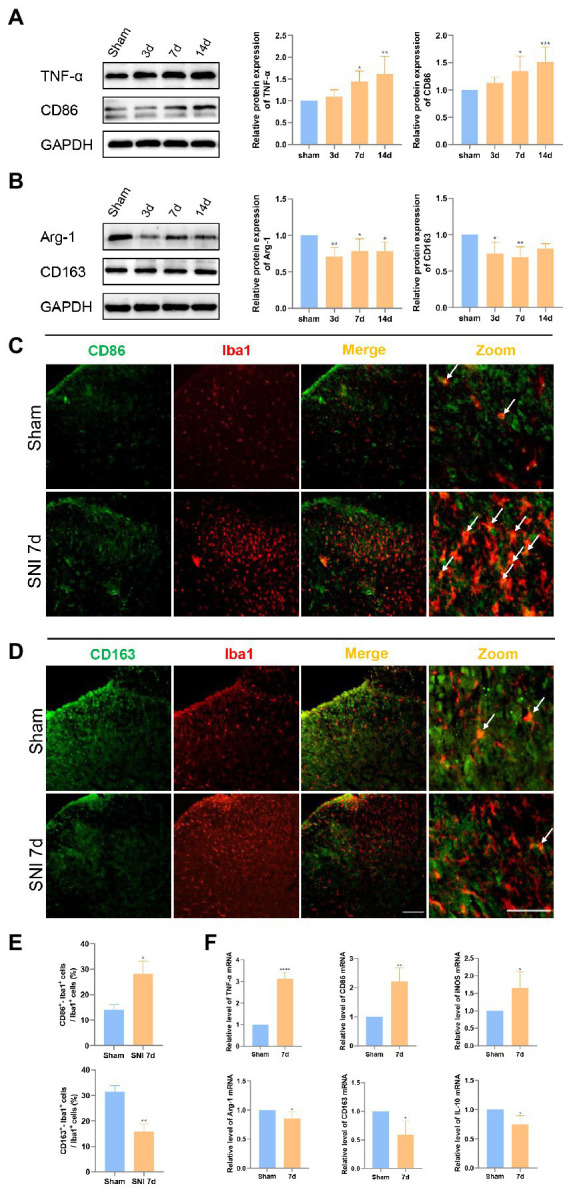
Microglia trended to the pro-inflammation stage after SNI. **(A)** Western blot showed that the expression of TNF-α and CD86 was significantly increased in the spinal dorsal cord at 7 and 14 days after SNI. **(B)** The expression of Arg-1 was decreased in the spinal dorsal cord at 3, 7, and 14 days, and the expression of CD163 was significantly decreased at 3 and 7 days after SNI. (*p < 0.05, **p < 0.01, ***p < 0.001 compared with sham group, *n* = 6 in each group). **(C)** Double immunostaining of CD86 (green) and Iba1 (red) in the spinal dorsal horn. The arrows show the representative merge of CD86 and Iba1. **(D)** Double immunostaining of CD163 (green) and Iba1 (red) in the spinal dorsal horn. The merge of CD163 and Iba1 is represented by arrows. Scale bars, 100 μm for lower magnification and 50 μm for higher magnification. **(E)** Histograms are quantifications of the percentage of CD86^+^−Iba1^+^ cells and CD163^+^−Iba1^+^ cells. Results of each group were obtained from three rats, and three micrographs from each sample. The average values of each sample were taken for statistics. (*p < 0.05, **p < 0.01 compared with sham group). **(F)** The expression of TNF-α mRNA, CD86, and iNOS mRNA was significantly increased and Arg-1, CD163, and IL-10 mRNA was decreased in the spinal dorsal cords of SNI 7d rats (*p < 0.05, **p < 0.01, ***p < 0.001 compared with sham group, *n* = 4 in each group).

In order to better describe the changes in microglia after SNI, the following experiments were performed. The western blot results revealed that when compared with the sham group, the protein content of TNF-α and CD86 increased, with a significant difference on days 7 and 14 after SNI. The protein content of Arg-1 and CD163 decreased, which was statistically significant on days 3 and 7 (Figures 2A,B). Therefore, it can be concluded that microglia may play a role in neural repair and promotion of homeostasis in the non-acute phase of SNI. In this study, we chose day 7 as the main time point of the study. At this time, microglia were functioning discrepantly between two groups. Meanwhile, microglia in the spinal dorsal horn mainly secreted proinflammatory factors, which further promoted the reaction of microglia.

According to the results of immunofluorescence, the proportion of CD86^+^/CD163^+^ microglia in the dorsal horn of the spinal cord was more intuitive on day 7 after SNI (Figures 2C,D). Compared with the sham group, the proportion of CD86^+^ Iba1^+^ cells/Iba1^+^ cells increased, and the proportion of CD163^+^ Iba1^+^ cells/Iba1^+^ cells decreased (Figure 2E). PCR also confirmed the same results. TNF-α, CD86 and iNOS mRNA levels increased, while homeostasis maintainers Arg-1, CD163 and IL-10 mRNA levels decreased (Figure 2F).

### PM226 alleviated mechanical hyperalgesia and cold hyperalgesia in rats

In order to explore the optimal dose of PM226 for intrathecal injection, three concentration gradients of 0.2, 2, and 20 nm were set according to previous experiments (Gomez-Canas et al., 2016). This time point was chosen given the relative stability of rat hyperalgesia on day 3 of SNI. Rats were divided into four groups for a single intrathecal administration of 15 μL (Supplementary Figure 2A). After administration, the mechanical withdrawal threshold was recorded at 0, 2, 4, 6, 8, 12, 24, and 48 h. As shown in Supplementary Figure 2B, compared with the vehicle group, there was no significant difference in the PM226 0.2 nm group, whereas the MWT of the PM226 2 nm group began to rise at 2 h, reached the maximum at 12 h, and decreased at 24 h and 48 h. The MWT of the PM226 20 nm group remarkably increased at 6 h and for 48 h with a similar trend to that of the PM226 2 nm group. In conclusion, a single intrathecal injection of PM226 2 nm and 20 nm can reduce the mechanical sensitivity after SNI. Considering the saving of drugs and the possible unknown side effects of large doses, 2 nm was selected as the concentration used in the next step.

**Figure 3:**
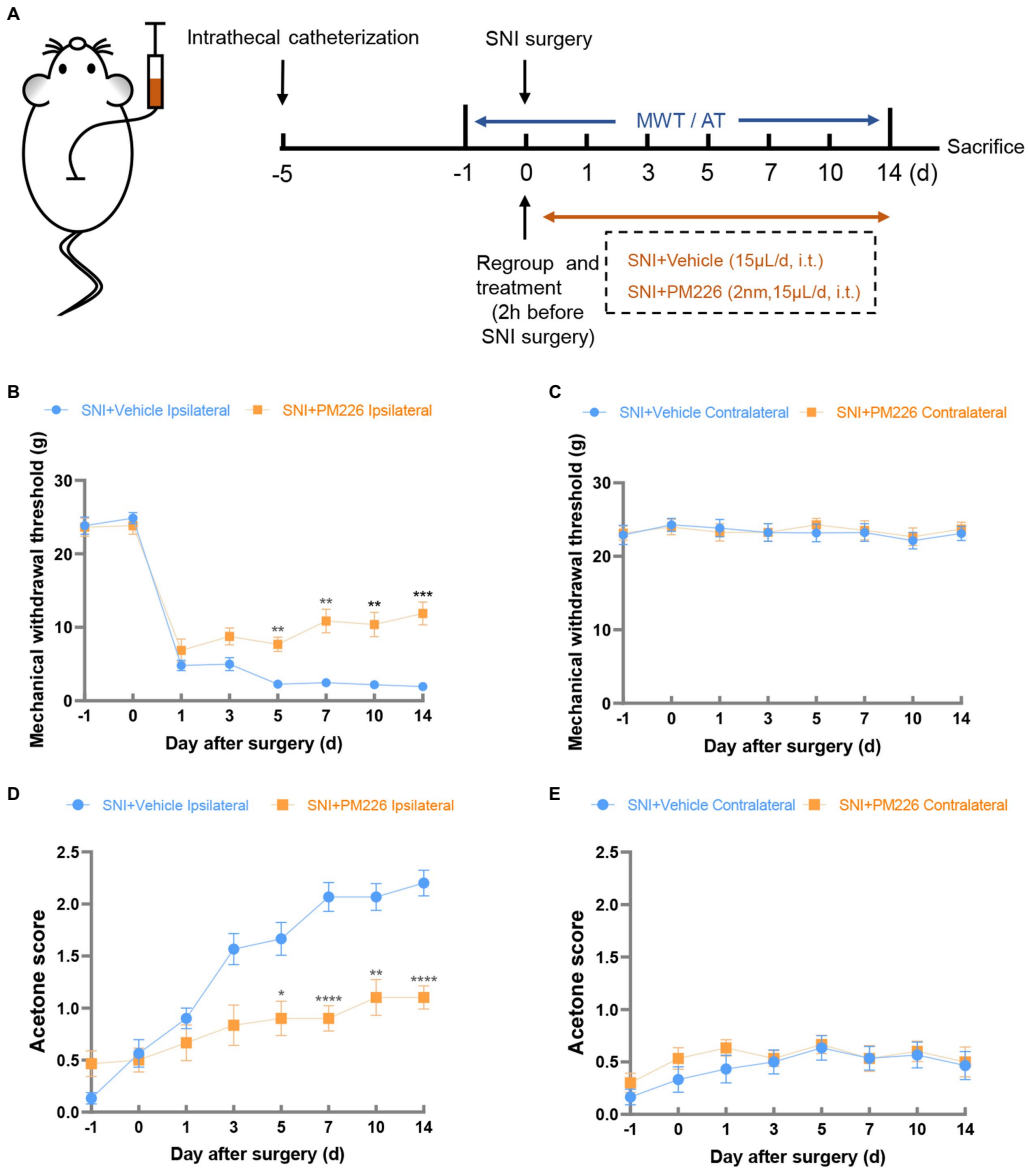
The mechanical and cold hyperalgesia were alleviated in the ipsilateral paws of rats after PM226 treatment. **(A)** Timeline of the experimental process. **(B,D)** Continuous intrathecal administration of PM226 improved the MWT **(B)** and decreased the acetone score **(D)** in the ipsilateral paws **(C,E)**. There was no significant difference in the MWT and acetone score between the two groups after PM226 treatment (*p < 0.05, **p < 0.01, ***p < 0.001, ****p < 0.0001 compared with SNI + Vehicle group, *n* = 10 in each group). MWT, mechanical withdrawal threshold; AT, acetone test.

In a further experiment, intrathecal catheterization on rats was performed, and the baseline value (−1d) after 5 days of recovery was measured. As mentioned above, PM226 at 2 nm was effective 2 h after administration, and we recorded the MWT, and acetone score 2 h after intrathecal injection of PM226. After the agonist took effect, the SNI surgery was performed. Then, administration was given at the same time point every day, and changes in hyperalgesia on days 1, 3, 5, 7, 10 and 14 were measured (Figure 3A). Compared with the SNI + Vehicle group, the MWT of the ipsilateral hind paw in the SNI + PM226 group increased significantly on day 5 (Figure 3B), and the acetone score decreased significantly (Figure 3D), which was maintained for 14 days. Continuous intrathecal administration of PM226 had no significant effect on the contralateral hind paw, which showed that the MWT and acetone score of the contralateral hind paw in the SNI + PM226 group were not significantly different from those in the SNI + Vehicle group (Figures 3C,E).

### PM226 blocked the proinflammatory process and promoted microglia to be beneficial

**Figure 4:**
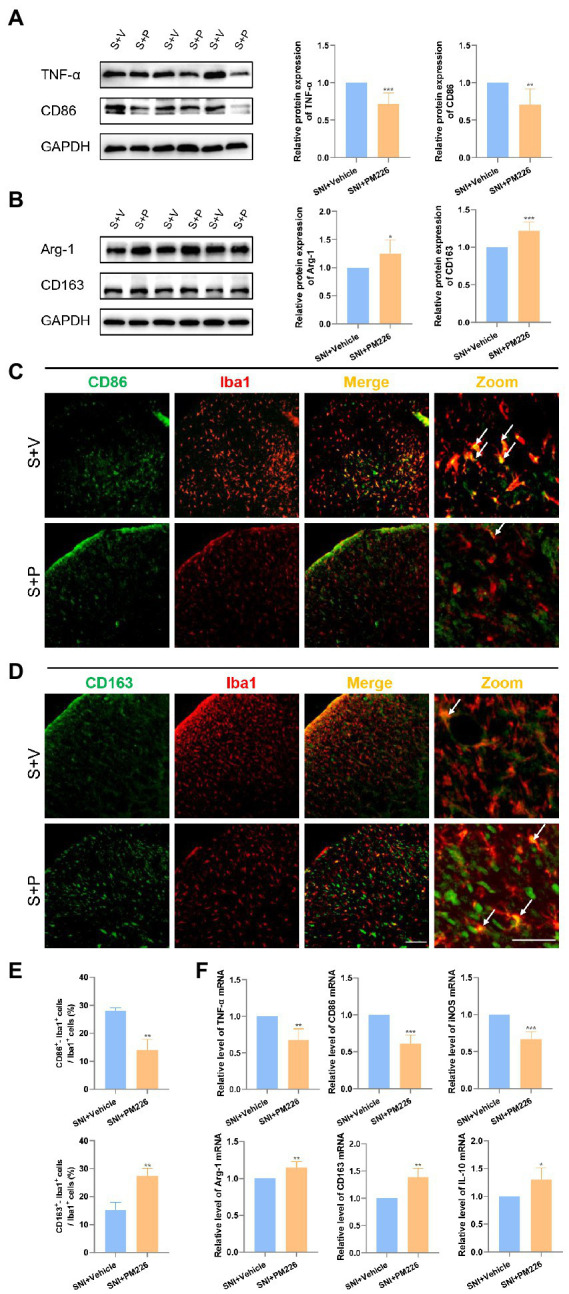
PM226 blocks the proinflammatory process and promotes microglia to be beneficial. **(A)** Western blot showed that the expression of TNF-α and CD86 was significantly decreased in the spinal dorsal cord after intrathecal administration for 7 days. **(B)** The expression of Arg-1 and CD163 was significantly increased in the spinal dorsal cord after intrathecal administration for 7 days. (*p < 0.05, **p < 0.01, ***p < 0.001 compared with SNI + Vehicle group, *n* = 6 in each group). **(C)** Double immunostaining of CD86 (green) and Iba1 (red) in the spinal dorsal horn. Arrows showed the representative merge of CD86 and Iba1. **(D)** Double immunostaining of CD163 (green) and Iba1 (red) in the spinal dorsal horn. Arrows showed the representative merge of CD163 and Iba1. Scale bars, 100 μm for lower magnification and 50 μm for higher magnification. **(E)** Histograms are quantifications of the percentage of CD86^+^−Iba1^+^ cells and CD163^+^−Iba1^+^ cells. Results for each group were obtained from three rats, and three micrographs from each sample. The average values of each sample were taken for statistics. (**p < 0.01 compared with SNI + Vehicle group). **(F)** The expression of TNF-α mRNA, CD86, and iNOS mRNA was significantly decreased and Arg-1, CD163, and IL-10 mRNA was increased in the spinal dorsal cords after intrathecal administration of PM226 for 7 days (*p < 0.05, **p < 0.01, ***p < 0.001 compared with SNI + Vehicle group, *n* = 4 in each group). S + V, SNI + Vehicle group; S + P, SNI + PM226 group.

Western blot results showed that compared with the SNI + Vehicle group, the protein expression of TNF-α and CD86 decreased while the expression of Arg-1 and CD163 protein increased (Figures 4A,B). It can be seen intuitively from the immunofluorescence pictures that after treatment, the double labeling of CD86 and Iba1 decreased (Figure 4C), while that of CD163 and Iba1 increased in the spinal dorsal horn (Figure 4D), the ratio of CD86^+^ Iba1^+^/Iba1^+^ decreased, and the ratio of CD163^+^ Iba1^+^ cells/Iba1^+^ cells increased significantly (Figure 4E). The PCR results were consistent with this, and the mRNA levels of TNF-α, CD86 and iNOS increased, while the levels of Arg-1, CD163 and IL-10 decreased (Figure 4F). In the immunofluorescence staining picture, it can also be observed that the Iba1 signal was significantly attenuated after the administration of PM226 (Figures 4C,D). The results showed that because of the plasticity of microglia, PM226 contributes to promoting microglia from the proinflammatory stage to regulatory stage, avoiding further over-reaction of microglia, and accelerating the body into the nerve recovery stage.

### PM226 suppressed P2X7 in the spinal dorsal horn of rats

**Figure 5:**
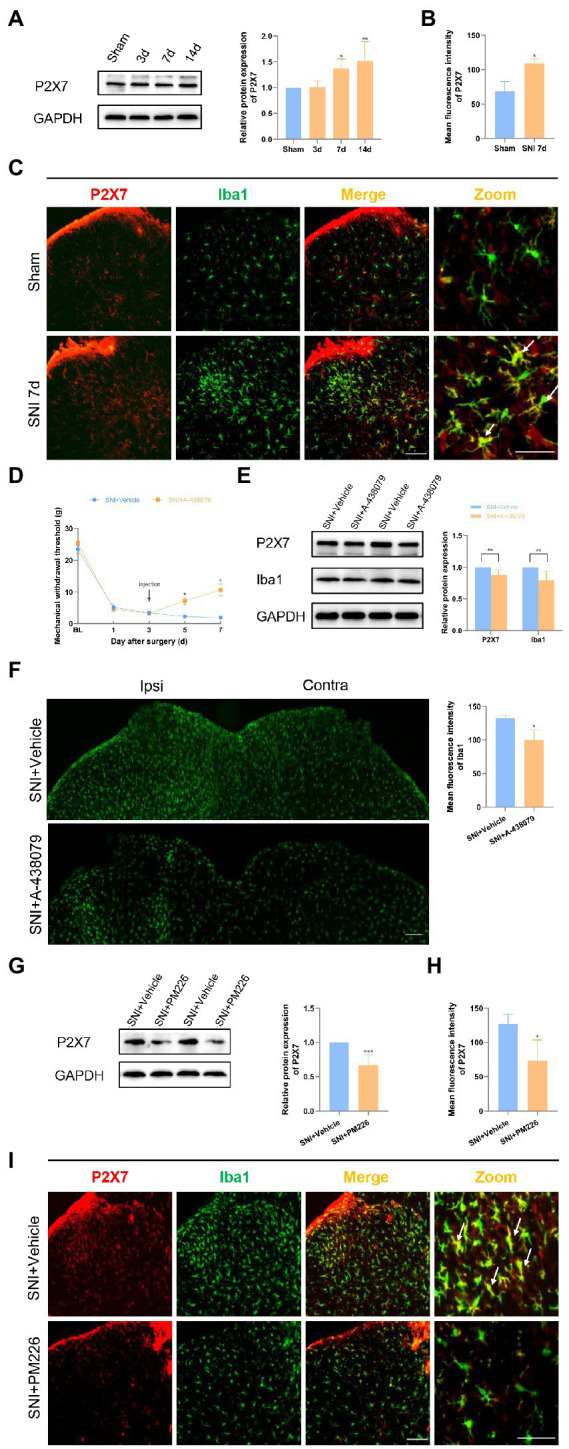
PM226 suppressed P2X7 in the rat spinal dorsal horn. **(A)** Western blot showed that the expression of P2X7 increased with time, and significantly increased on days 7 and 14 after SNI. (*p < 0.05, **p < 0.01 compared with sham group, *n = 6* in each group). **(B)** The mean fluorescence intensity of P2X7 (**C**, red) in the ipsilateral spinal dorsal horn of the sham and SNI 7d group. Results for each group were obtained from three rats, and three micrographs from each sample. The average values of each sample were taken for statistics. (*p < 0.05, compared with sham group). **(C)** Double immunostaining of P2X7 (red) and Iba1 (green) in the dorsal horn of the spinal cord. Arrows showed the representative merge of P2X7 and Iba1. Scale bars, 100 μm for lower magnification and 50 μm for higher magnification. **(D)** The administration of A-438079 on day 3 improved the MWT of the ipsilateral paws on days 5 and 7 (*p < 0.05 compared FIGURE 5 (Continued)with SNI + Vehicle group, *n* = 8 in each group). **(E)** The IBA1 protein level and P2X7 protein level in the spinal cord both decreased after A-438079 administration (**p < 0.01 compared with SNI + Vehicle group, *n* = 6 in each group). **(F)** The mean fluorescence intensity of Iba1 of ipsilateral spinal dorsal horn in the SNI + A-438079 group was significantly lower than that in the SNI + Vehicle group. Results of each group were obtained from three rats, and three micrographs from each sample. The average values of each sample were taken for statistics (*p < 0.05 compared with SNI + Vehicle group). **(G)** The protein expression of P2X7 was significantly decreased in the spinal dorsal cord after intrathecal administration of PM226 for 7 days. (***p < 0.001 compared with SNI + Vehicle group, *n* = 6 in each group). **(H)** The mean fluorescence intensity of P2X7 (**F**, red) in the ipsilateral spinal dorsal horn after intrathecal administration for 7 days. Results of each group were obtained from three rats, and three micrographs from each sample. The average values of each sample were taken for statistics. (*p < 0.05 compared with SNI + Vehicle group). **(I)** Double immunostaining of P2X7 (red) and Iba1 (green) in the spinal dorsal horn after intrathecal administration. Arrows showed the representative merge of P2X7 and Iba1. Scale bars, 100 μm for lower magnification and 50 μm for higher magnification.

A certain association between P2X7 and the progression of NP has been demonstrated in previous related studies (Chessell et al., 2005; Hu et al., 2020). P2X7 observations were performed in the dorsal horn of rat spinal cord. The P2X7 protein level significantly increased on days 7 and 14 after SNI surgery (Figure 5A). The immunofluorescence images of the spinal cord dorsal horn intuitively revealed this change in P2X7. Moreover, it could be seen that P2X7 and Iba1 were significantly co-labeled (Figures 5B,C).

The P2X7 inhibitor A-438079 was used to confirm the important role of P2X7 in neuropathic pain (NP) (Hu et al., 2020). Since the rat hyperalgesia tended to stabilize on day 3 after SNI, the vehicle, or P2X7 inhibitor, was administered to each group through the intrathecal tube after the pain behavioral test. Mechanical hyperalgesia was alleviated in the SNI+ A-438079 group compared to the SNI + Vehicle group on days 5 and 7 (Figure 5D). According to the WB results, it can be seen that the Iba1 protein level and P2X7 protein level in the spinal cord both decreased after A-438079 administration (Figure 5E). According to the immunofluorescence images, it can be found that the mean fluorescence intensity of Iba1 in the ipsilateral spinal dorsal horn in the SNI + A-438079 group was significantly lower than that in the SNI + Vehicle group (Figure 5F). The above results indicate that P2X7 plays a key role in the process of NP generation. In other words, blocking P2X7 in the early stage of NP (day 3) can produce the analgesic effect.

According to a prior study, CB2R and P2X7 may play a synergistic role in the regulation of neuroglial cells (Freitas et al., 2019). The relationship between the two was also explored in the NP model. After treatment with PM226, a CB2R agonist, the P2X7 protein level was significantly reduced (Figure 5G), the mean fluorescence intensity was weakened (Figure 5H), and the co-expression of P2X7 with Iba1^+^ cells was also decreased (Figure 5I). Based on these results, it is possible to concluded that CB2R activation can inhibit P2X7 and produce the therapeutic effect on NP.

## Discussion

The generation of NP is a complicated process, in which microglia play a crucial role. After direct or indirect injury of the primary afferent nerve, immune cells such as peripheral macrophages and central resident microglia will respond immediately by accumulating around neurons through proliferation, infiltration or migration, and release proinflammatory factors to act on nociceptors, thereby causing peripheral sensitization (Domoto et al., 2021). It can be observed that 7 days after SNI, the microglia in the spinal dorsal horn were significantly reactive, which manifests as increased numbers, larger cell bodies, and shorter branches. After SNI, microglia can be overactive through the ASK-1/JNK/P-38 pathway and tend toward a harmful stage (Zhang et al., 2022). To investigate the alteration of microglia during NP development, the characteristics of microglia in the spinal dorsal horn were observed on days 3, 7, and 14 of SNI rats. TNF-α and CD86, proinflammatory molecules, increased significantly on days 7 and 14, while homeostatic molecules Arg-1 and CD163, decreased significantly on days 3 and 7 compared to the sham group, and CD163 slightly recovered on day 14. It indicates that in the different stages of NP, microglia have the plasticity to function diversely to become harmful or beneficial. Day 7 was chosen as the main time point of this study. At this time, proinflammatory factors such as TNF-α and iNOS were secreted by microglia in the dorsal horn, while anti-inflammatory factors such as Arg-1 and IL-10 were relatively decreased. The immunofluorescence co-labeling results all indicated that SNI induced microglia to turn to different stages that were opposite to homeostasis.

For centuries, cannabinoid preparations have been used to treat chronic pain by producing adverse psychiatric symptoms and the potential for abuse, which has limited their widespread clinical use (Le Boisselier et al., 2017; O’Hearn et al., 2017). 7-(1,1dimethylheptyl)-4,4-dimethyl-9-methoxychromeno[3,4-d] isoxazole (PM226) is a novel and highly selective CB2R agonist, which showed good neuroprotective effects *in vivo* and *in vitro* experiments (Gomez-Canas et al., 2016). However, its therapeutic effect on NP has not been confirmed. This study can contribute to a certain extent to promote the clinical application of derivatives related to the endocannabinoid system. This study found that a single intrathecal injection of the CB2R specific agonist PM226 can relieve hyperalgesia, with the effect peaking at 12 h and decreasing from 12 to 48 h, but the difference remained statistically significant. In addition, continuous intrathecal administration daily from the day of SNI surgery can effectively reduce hyperalgesia on day 5. It is well known that microglia play a key role in the initiation of mechanical and thermal hyperalgesia and cooperate with peripheral monocytes to facilitate the transition from acute to chronic pain following peripheral nerve damage in NP rodent models (Peng et al., 2016). Putting CB2R into an activated state at the time of nerve damage, namely at the early stage of pain, is effective in preventing the chronic progression of pain. The role of CB2R in the brain remains controversial. Although its expression is restricted to specific neurons, it is abundant in microglia and astrocytes, which act as key regulators of inflammatory and nociceptive responses, controlling immune cell activation and migration (Palazuelos et al., 2009; Narouze, 2021; Hu et al., 2022). Earlier research mainly concentrated on the impact of CB2R agonists on the activation of microglia (Racz et al., 2008; Wu et al., 2019; Wang et al., 2020). This study found that intrathecal administration of PM226 could regulate the function of microglia from the destroyer to the protector, which implies that CB2R activation plays a neuroprotective rather than immunosuppressive role in alleviating NP-induced hyperalgesia.

At the acute phase after nerve damage, microglia secrete a series of proinflammatory factors such as TNF-α, IL-1 β, IL-6, and iNOS, which further stimulate microglia and make them overactive (Lan et al., 2017). PM226 treatment altered the stages of microglia by blocking the secretion of proinflammatory factors and promoting the increase of anti-inflammatory factors, which exhibited the neuroprotective role of CB2R. Some studies have proposed that the complete dualistic microglia classification of M1/M2 is not applicable *in vivo* because it is based on *in vitro* studies (Devanney et al., 2020). For this, we used multiple markers, CD86 and CD163 are on the cell surface, TNF-α, Arg-1 and IL-10 are secreted, while iNOS is cellular content. Changes in these molecules, to a certain extent, depict the functional alteration of microglia. The processes *in vivo* and *in vitro* can be very similar. As for more advanced guidance, it is recommended to describe microglia using as many layers of complexity as possible: morphology, motility, omics, and function, consistently placing them into a species and spatiotemporal context (Paolicelli et al., 2022). This is also the direction for follow-up research.

In order to avoid the effect of estrogen on pain perception, we tend to select male animals to perform NP models. However, it is undeniable that microglia differ in quantity, phenotype and transcriptome between male and female. Male rodents’ microglia are more active in certain brain areas than female rodents’ microglia, have higher antigen-presenting ability, and seem to have a higher response potential to ATP (Guneykaya et al., 2018; Villa et al., 2018; Han et al., 2021). In repeated inflammatory pain, β-caryophyllene (BCP), binding to the CB2R and acting as a full agonist, alleviated the pain in both male and female rats, but there were behavioral differences at different stages at the experiment (Ceccarelli et al., 2020). Since activational and maybe organizational effects of gonadal hormones leads to different responses to cannabinoids between male and female (Diaz et al., 2009; Craft et al., 2013). In clinical treatment, we tend to apply medication to male and female patients indiscriminately. However, we believe it is useful to take the biological sex specificity of microglia into clinical consideration.

P2X7, a member of the purinergic receptor family, is mainly expressed by immune cells. In addition, when it activates, inflammatory factors such as IL-1β and IL-6 will be released (Chessell et al., 2005). According to an *in vitro* investigation, the presence of a CB2R agonist can enhance the effect of ATP on P2X7 activation during the formation of chick embryonic retinal glial cells. Furthermore, the presence of P2X7 is essential for CB2R agonists to function (Freitas et al., 2019). To explore whether there is an interaction between CB2R and P2X7 during NP, the expression of P2X7 after SNI and after PM226 treatment was observed. It can be found that P2X7 content was increased, while microglia were significantly activated at 7 and 14 days after SNI. Studies have shown that P2X7 inhibitors can reduce hyperalgesia and reduce Iba1 and GFAP levels (Hu et al., 2020; Campagno et al., 2021). However, the content of P2X7 decreased and the co-labeling with Iba1 decreased after PM226 administration in the spinal dorsal horn. Therefore, it can be concluded that PM226 can downregulate the elevated P2X7 after SNI, thereby preventing the overactivation of microglia. Whether CB2R agonists regulate P2X7 by regulating ATP or through some molecular loop is a question worthy of further discussion.

The findings of this study are as follows: In SNI-induced NP, microglia overactivation and the pro-inflammation stage are the causes of hyperalgesia in rats. Intrathecal administration of PM226 can significantly suppress this inflammatory reaction and promote microglia into a beneficial stage. Furthermore, activation of CB2R can downregulate P2X7, thereby inhibiting further activation of microglia and production of inflammatory factors. This study introduces a novel concept and provide support for CB2R agonists in the treatment of NP.

## Conclusion

Continuous intrathecal injection of CB2R agonist PM226 can alleviate the mechanical and cold hyperalgesia in rats after SNI, which is related to altering microglial stages from harmful to beneficial. Meanwhile, PM226 also suppresses P2X7, which prevents microglia from overreacting, thus achieving the effect of reducing NP.

## Data availability statement

The original contributions presented in the study are included in the article/Supplementary material, further inquiries can be directed to the corresponding authors.

## Ethics statement

The animal study was reviewed and approved by the Ethics Committee of 2nd Affiliated Hospital of Zhengzhou University (No. 2022055).

## Author contributions

YZ and YX is responsible for most of the experimental design, data collection and analysis, and manuscript writing. JY carried out western blot experiment and result analysis. WW, ZY, and MY conducted behavioral tests and assisted in completing the other experiments. YW completed the review and statistical analysis of the final data. QB and ZL designed the experiments, supervised the overall study, directed the writing, and reviewed the final manuscript. All authors contributed to the article and approved the submitted version.

## Funding

This work was supported by grants from the Henan province young and middle-aged health science and technology innovation outstanding young talent training project (No. YXKC2021018) and the National Natural Science Foundation of China Youth Project (No. 82001191).

## Conflict of interest

The authors declare that the research was conducted in the absence of any commercial or financial relationships that could be construed as a potential conflict of interest.

## Publisher’s note

All claims expressed in this article are solely those of the authors and do not necessarily represent those of their affiliated organizations, or those of the publisher, the editors and the reviewers. Any product that may be evaluated in this article, or claim that may be made by its manufacturer, is not guaranteed or endorsed by the publisher.

## Supplementary material

SUPPLEMENTARY FIGURE S1 SNI induced the mechanical and cold hyperalgesia in the ipsilateral paws of rats. **(A)** SNI significantly reduced the MWT and improved the acetone score in the ipsilateral hind paws of the SNI group (*****p*<0.0001 compared with contralateral hind paws). **(B)** There was no significant difference in the pain sensitivity of the contralateral hind paws between the sham and SNI groups (*n* = 12 in each group).

SUPPLEMENTARY FIGURE S2 Test of the optimum concentration of intrathecal administration of PM226. **(A)** Timeline and grouping information of the experimental process. **(B)** Time course of mechanical hypersensitivity changes after a single injection of different concentrations of PM226 (**p*<0.05, ***p*<0.01, ****p*<0.001, *****p*<0.0001 compared with SNI+Vehicle group, *n* = 8 in each group).
