# Supplementary figures and images for: Spinal cannabinoid receptor 2 activation alleviates neuropathic pain by regulating microglia and suppressing P2X7 receptor
Source: Front Mol Neurosci. 2023 Mar 8;16:1061220. doi: 10.3389/fnmol.2023.1061220 (PMC10030493; doi:10.3389/fnmol.2023.1061220)

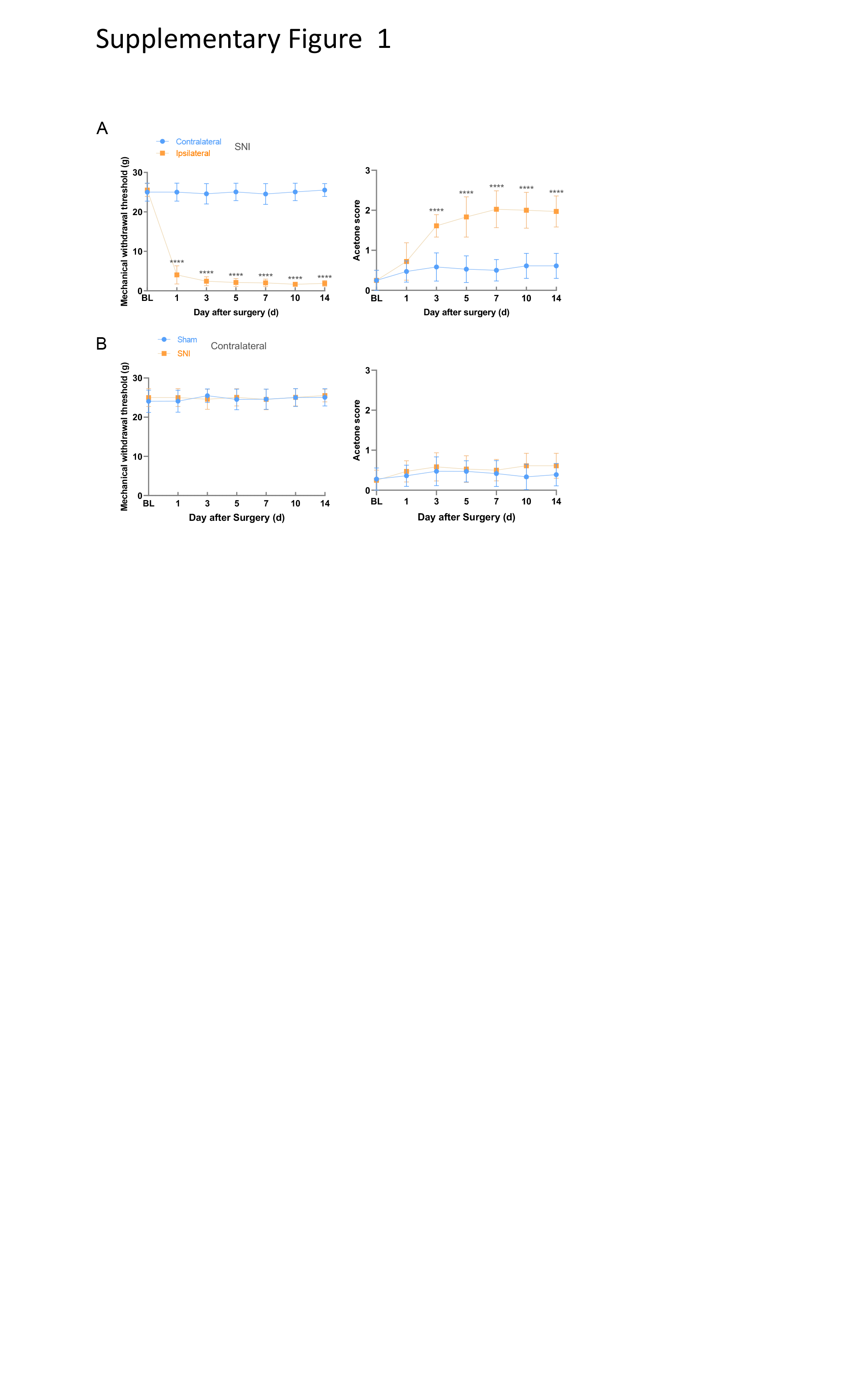

Supplement: SUPPLEMENTARY FIGURE S1 — SNI induced the mechanical and cold hyperalgesia in the ipsilateral paws of rats. (A) SNI significantly reduced the MWT and improved the acetone score in the ipsilateral hind paws of the SNI group (****p<0.0001 compared with contralateral hind paws). (B) There was no significant difference in the pain sensitivity of the contralateral hind paws between the sham and SNI groups (n = 12 in each group). [file Image_1.tif]

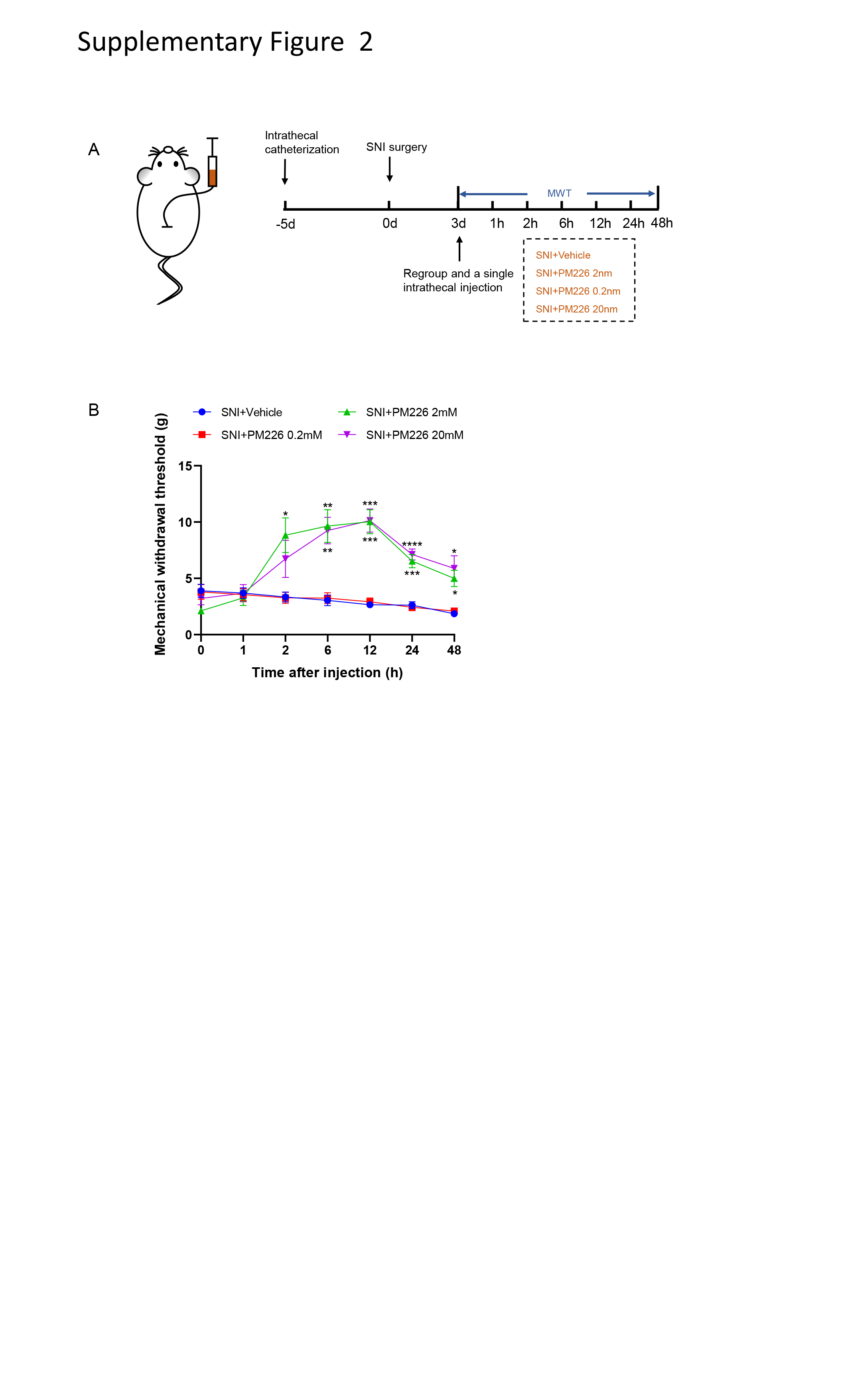

Supplement: SUPPLEMENTARY FIGURE S2 — Test of the optimum concentration of intrathecal administration of PM226. (A) Timeline and grouping information of the experimental process. (B) Time course of mechanical hypersensitivity changes after a single injection of different concentrations of PM226 (*p<0.05, **p<0.01, ***p<0.001, ****p<0.0001 compared with SNI+Vehicle group, n = 8 in each group). [file Image_2.tif]
